# Genetic Variants of *BMP2* and Their Association with the Risk of Non-Syndromic Tooth Agenesis

**DOI:** 10.1371/journal.pone.0158273

**Published:** 2016-06-30

**Authors:** Yun Lu, Yajing Qian, Jinglu Zhang, Miao Gong, Yuting Wang, Ning Gu, Lan Ma, Min Xu, Junqing Ma, Weibing Zhang, Yongchu Pan, Lin Wang

**Affiliations:** 1 Jiangsu Key Laboratory of Oral Diseases, Nanjing Medical University, Nanjing, China; 2 Department of Orthodontics, Affiliated Hospital of Stomatology, Nanjing Medical University, Nanjing, China; 3 Department of Orthodontics, College of Stomatology, Dalian Medical University, Dalian, China; 4 Orofacial Pain and TMD Research Unit, Institute of Stomatology, Affiliated Hospital of Stomatology, Nanjing Medical University, Nanjing, China; International Centre for Genetic Engineering and Biotechnology, ITALY

## Abstract

Non-syndromic tooth agenesis (or non-syndromic congenitally missing tooth) is one of the most common congenital defects in humans affecting the craniofacial function and appearance. Single nucleotide polymorphisms (SNPs) have been associated with an individual’s susceptibility to these anomalies. The aim of the present study was therefore to investigate the roles of the potentially functional SNPs of *BMP2* in the occurrence of tooth agenesis. Overall, four potentially functional SNPs of *BMP2* (rs15705, rs235768, rs235769 and rs3178250) were selected, and their associations with the susceptibility of tooth agenesis were evaluated in a case-control study of 335 non-syndromic tooth agenesis cases and 444 healthy controls. The SNPs rs15705 and rs3178250 were found to be associated with an individual’s risk of tooth agenesis (*P* = 0.046 and *P* = 0.039, respectively). Both SNPs showed an increased risk of mandibular incisor agenesis (rs15705, AA/AC *vs*. CC = 1.58, 95% CI = [1.06–2.34], *P* = 0.024; rs3178250, TT/TC *vs*. CC = 1.60, 95% CI = [1.08–2.37], *P* = 0.020). Bioinformatics analysis indicated that these two SNPs located at the 3’-untranslated region (3’-UTR) of *BMP2* might alter the binding ability of miR-1273d and miR-4639-5p, respectively, which was confirmed by luciferase activity assays in the 293A and COS7 cell lines (*P* < 0.001 in 293A and *P* < 0.01 in COS7 for miR-1273d; and *P* < 0.001 in both cells for miR-4639-5p). Furthermore, *BMP2* mRNA expression decreased after transfecting either miR-1273d or miR-4639-5p into these two cell lines (*P* < 0.01 in 293A and *P* < 0.001 in COS7 for miR-1273d, and *P* < 0.01 in both cell lines for miR-4639-5p). Taken together, our findings indicate that rs15705 and rs317250 are associated with the susceptibility of non-syndromic tooth agenesis by possibly affecting miRNAs and mRNA interaction.

## Introduction

Tooth agenesis (or congenitally missing tooth) is one of the most common congenital defects in humans, and it may affect individual’s appearance, chewing ability, speech, facial development and overall health. The average worldwide prevalence of tooth agenesis (excluding the third molars) is 6.4%, with the highest prevalence in Africans, followed by Europeans, Asians, Australians, and then North Americans, Latin Americans and Caribbeans [1]. In the Chinese population, a prevalence of 5.89% in the general population and 7.48% in orthodontic subjects has been reported, with the second mandibular premolars and the maxillary lateral incisors most frequently affected [2]. However, for Caucasians, the most common congenitally missing teeth (excluding the third molars) are the second mandibular premolars, the maxillary lateral incisors, and the maxillary second premolars [3].

Tooth agenesis can be classified into two main types: syndromic and non-syndromic. Syndromic tooth agenesis refers to complex developing syndromes associated with a congenitally missing tooth or teeth, such as non-lethal Raine syndrome [4], cleft lip and palate [5] and HATS syndrome [6]. In contrast, non-syndromic tooth agenesis typically involves a congenitally missing tooth in an isolated form without any other major birth defects.

The development of non-syndromic tooth agenesis has resulted from multiple factors [7]. Genetic factors may play a vital role, as suggested by the substantial prevalence variation among different ethnic groups, twin analyses as well as family studies [8–10].

SNPs are single DNA sequence variations occurring in the genome, and these are the most common form of genetic variation among humans, accounting for more than 90% of the known variation [11]. They are associated with various types of human traits, including non-syndromic tooth agenesis. For instance, Haga S. et al. conducted a genome-wide association study and found strong association between rs1469622 and the third molar agenesis [12]. Furthermore, *WNT10A* variations contributed to severity of tooth agenesis in Song’s study [13]. However, these identified SNPs far from clearly elucidated the genetic susceptibility of non-syndromic tooth agenesis [14]. Therefore, to better understand the etiology of non-syndromic tooth agenesis, other susceptible SNPs need to be identified.

The bone morphogenetic protein (*BMP*) family, comprising an extensive group of phylogenetically conserved growth factors, such as *BMP2*, *BMP4* and *BMP7*, plays an important role during tooth development. For instance, the *BMP4* expression pattern coincides with the bud-to-cap stage transition in tooth development [15]. Our previous study found that SNP rs17563 of *BMP4* is associated with non-syndromic tooth agenesis [16].

*BMP2*, another important member of the *BMP* family, is known to be involved in regulating tooth initiation and shape development and can induce human tooth germ cells to differentiate into odontogenic and osteogenic cells [17–18]. It is mainly localized to the developing tooth buds, jawbone, and striated and smooth muscle in human embryos [19]. In mice, *BMP2* expressed in the presumptive dental epithelium [20], could result in the arrest of tooth development after knockdown [21]. *In vitro*, *BMP2* regulates the odontogenic differentiation of dental pulp cells and controls the mineralization processes of the dentin formation [22].

In summary, these findings demonstrate the important roles of *BMP2* during tooth development. Previous studies have tried to investigate the genetic contributions of *BMP2* in the family or in sporadic non-syndromic tooth agenesis, but the results were not consistent. In the present study, we designed an ongoing hospital-based case-control study and selected four potentially functional SNPs of *BMP2* to explore their associations with the risk of non-syndromic tooth agenesis.

## Materials and Methods

### Human Subjects

This study consisted of 335 non-syndromic tooth agenesis cases and 444 healthy controls from the Affiliated Stomatological Hospital of Nanjing Medical University and Nanjing First People’s Hospital between October 2005 and March 2014 [16]. All cases eligible with at least one missing permanent tooth (the third molar excluded) were recruited with the following exclusion criteria, which were also described in our previous study: (1) cleft lip and/or palate or other syndromes, such as non-lethal Raine syndrome [4] or HATS syndrome [6], (2) tooth absence due to trauma, periodontal disease, extraction, caries or fused tooth, and (3) the second molar germ absence in the mixed dentition stage. All controls had complete dentition, including their third molars, without any other craniofacial deformity. Approximately 2 ml of whole blood was collected from all subjects using an anticoagulant dying tube with EDTA, and the blood sample was separated immediately into plasma and cellular fractions by centrifugation at 3,000 rpm for 6 min at 4°C. According to the clinical and radiographic examination and the dental treatment history, two researchers assessed the cases and controls. All samples involved in the study were genetically unrelated members of the Chinese population. After informing the participants and their parents or guardians of the main objective and whole process of the study, written informed consent (as outlined in PLOS consent form) to participate in this study and to publish the case details were obtained from all participants. The present study was approved by the Institutional Review Board of Nanjing Medical University (PJ2004-030-001).

### Selection of potentially functional *BMP2* SNPs

The aim of the present study was to evaluate the associations between potentially functional SNPs of *BMP2* and the risks of non-syndromic tooth agenesis. The potentially functional SNPs of *BMP2* were selected from the dbSNP database (http://www.ncbi.nlm.nih.gov/snp/) and SNPinfo (http://snpinfo.niehs.nih.gov/) based on the following criteria: (1) a MAF (minor allele frequency) ≥ 5% in the Chinese population, and (2) a location in the 5’flanking regions, 5’-UTR, 3’-UTR, or coding regions leading to amino acid changes [23].

### DNA extraction and Genotyping

Genomic DNA was extracted from the samples by conventional methods with the QIAmp Blood kit (QIAGEN, Germany). Selected SNPs were genotyped according to the conventional TaqMan-MGB methodology on an ABI-Prism 7900 instrument (Applied Biosystems, Foster City, CA). The primers of the Taqman probes are listed in S1 Table. Two duplicates and one negative control (water) were chosen in the TaqMan assays for quality control. Two researchers reviewed the genotyping results independently in a blind manner. In addition, 10% samples were randomly selected for repeat analysis, and the results were 100% concordant. The call rate of the cases and controls is listed in S2 Table. DNA samples that failed to be genotyped were excluded from further analyses.

### Cell Culture

The 293A and COS7 cells were cultured in Dulbecco's Modified Eagle's Medium (DMEM; Gibco, Darmstadt, Germany) comprised of 10% fetal bovine serum (FBS), 100 U/ml penicillin and 100 mg/ml of streptomycin in a humidified atmosphere containing 5% CO_2_ at 37°C. The culture medium was replaced every other day.

### Luciferase reporter plasmid construction and luciferase reporter assay

The *BMP2* 3’-UTR region containing rs15705 (A/C) and rs3178250 (T/C) was synthesized wild type (rs15705 A allele and rs3178250 T allele, WT) and mutation type (rs15705 C allele and rs3178250 C allele, MT), and inserted between the restrictive sites *Xho*I and *Not*I of psiCHECK^TM^-2 vector (Promega, Madison, WI, USA). DNA sequencing was used to confirm the accuracy of the constructed plasmids (S1 Fig).

For the luciferase activity assay, 293A and COS7 cell lines were co-transfected with *BMP2* 3’-UTR luciferase reporter WT or MT plasmids and miR-1273d or miR-4639-5p mimics with Lipofectamine 2000 (Invitrogen, Carlsbad, CA, USA) for 4–6 h. The samples were then split by passive lysis buffer 24 h after transfection before being assayed for luciferase activity using a Dual-Luciferase Reporter Assay System (Promega). The ratio of Firefly luciferase to Renilla luciferase was calculated to evaluate the luciferase activity. Each plasmid experiment was replicated in triplicate with three duplication wells.

### miRNA Transfection and Real-time Quantitative PCR

293A and COS7 cells were transfected with miR-1273d mimics or miR-4639-5p mimics, respectively, with Lipofectamine 2000 (Invitrogen, Carlsbad, CA, USA) for 48 h, and split by TRIzol reagent (Invitrogen) according to the manufacturer’s protocol. cDNA synthesis was conducted with 1*μ*g total RNA processed with the cDNA synthesis kit (Takara, Shiga, Japan). Real-time quantitative PCR was performed using Power SYBR Green on a 7900 Real-Time PCR System (Applied Biosystems, Foster City, CA, USA). The primers were *BMP2-F*: *CCAGGTTAGTGACTCAGAACAC* and *BMP2-R*: *TCATCTTGGTGCAAAGACCTGC*. The expression levels of *BMP2* mRNA were normalized to the expression levels of the control gene *GAPDH*. Each plasmid transfection was performed in triplicate, with three duplication wells.

### Statistical Analysis

All tests were performed with SAS software (version 9.1; SAS Institute, Inc., Cary, NC, USA). Distributions in the gender and age were tested by Chi-Square test and Independent-Sample *t* test. The Hardy-Weinberg equilibrium (HWE) was assessed in the controls by a goodness-of-fit χ^*2*^ test (*P* > 0.05). The odds ratio (OR) and 95% confidence interval (CI) were used to estimate associations between the SNPs and the corresponding risk of tooth agenesis.

Linkage disequilibrium (LD) and haplotype analysis were assessed by Phase 2.1 software according to the *D′* and *r*^*2*^ values. Luciferase activity analysis and qPCR were calculated by Student′s *t* test and one-way analysis of variance (ANOVA).

## Results

### Characteristics of the samples

In the current case-control study, we enrolled 335 cases (mean age: 16.42 ± 6.57) and 444 controls (mean age: 17.05 ± 8.34). Selected characteristics of all samples are shown in S3 Table. The ages and genders of the participants were both well-matched between the cases and controls (*P* = 0.265 and *P* = 0.602, respectively).

**Fig 1:**
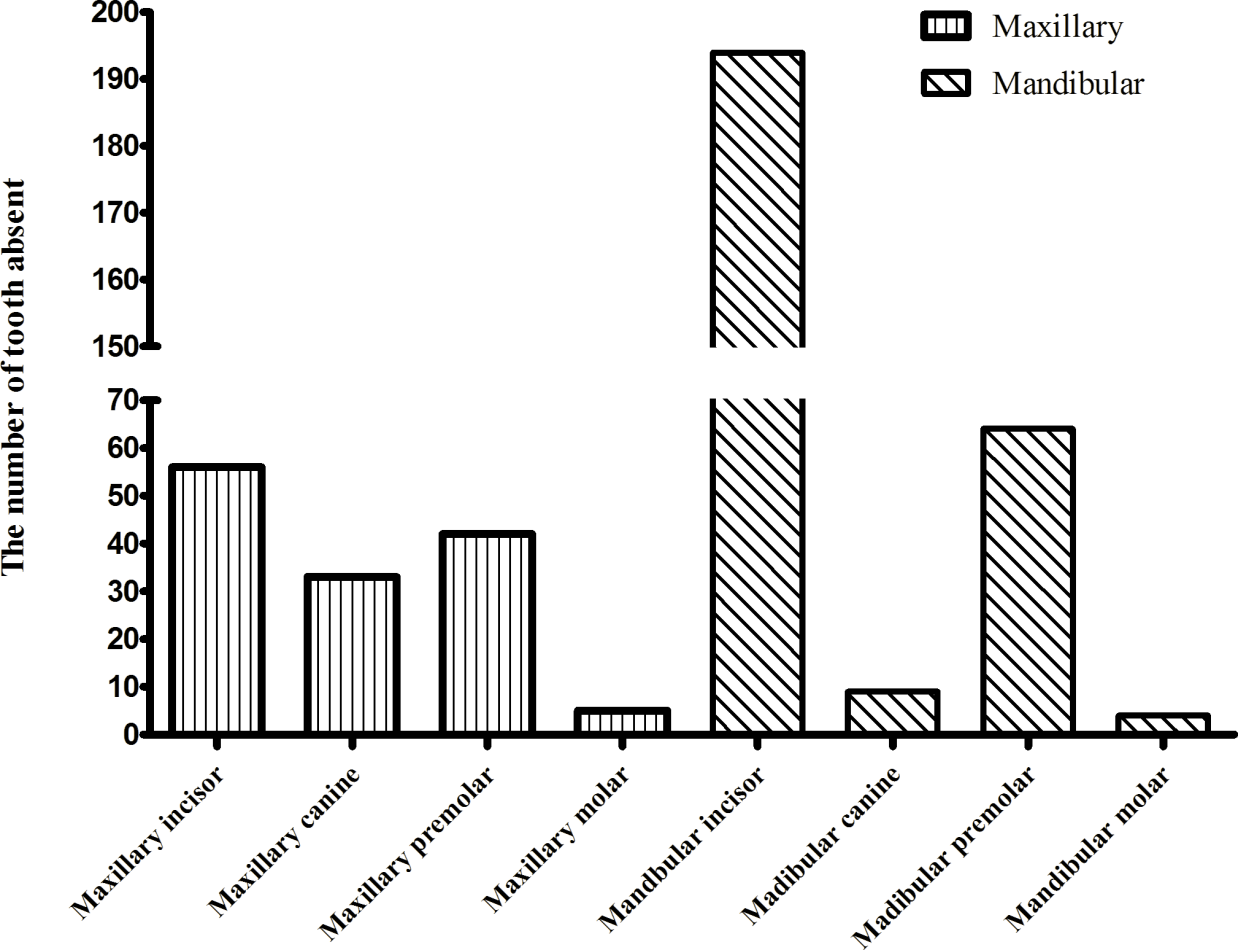
Distributions of congenitally missing tooth.

The distributions of the congenitally missing tooth among the cases are presented in Fig 1. The most commonly missing tooth was the mandibular incisor (N = 194), followed by the mandibular premolar (N = 64) and maxillary incisor (N = 56).

### SNPs identification and genotyping

According to the criteria used for the SNP selection, four SNPs (rs15705, rs235768, rs235769 and rs3178250) were selected for the present analysis. SNPs rs15705, rs235769 and rs3178250 were located in the 3’-UTR, while SNP rs235768 was located in the exon of *BMP2*. All SNPs were successfully genotyped with a call rate > 99% among the cases and controls. In addition, the genotype distributions of the four SNPs in the controls were all consistent with HWE with *P >* 0.05.

### Overall analysis between *BMP2* SNPs and the risk of tooth agenesis

The overall associations between the four SNPs (rs15705, rs235678, rs235679 and rs3178250) and the tooth agenesis risks are presented in Table 1. For each SNP, genotype and allele frequency, the *P* value for their distribution between cases and controls, genotype comparison (including co-dominant, dominant and recessive model) and allele comparison were provided.

**Table 1:** Associations between the four *BMP2* SNPs and tooth agenesis susceptibility.

Genotype	Controls	Cases	*P*^a^	Pattern	OR (95% CI) ^b^
	*N* = 444 (%)	*N* = 335 (%)			
rs15705 (A > C)				AC *vs*. AA	0.74 [0.53–1.03]
AA	120 (27.2)	103 (30.7)	**0.046**	CC *vs*. AA	1.12 [0.75–1.68]
AC	236 (53.5)	150 (44.8)		AC / CC *vs*. AA	0.84 [0.62–1.15]
CC	85 (19.3)	82 (24.5)		AA / AC *vs*. CC	1.36 [0.96–1.91]
C / A allele	406 (46.0)/ 476 (54.0)	314 (46.9)/ 356 (53.1)	0.744	C *vs*. A	1.03 [0.85–1.27]
rs235768 (T > A)				TA *vs*. TT	1.00 [0.74–1.36]
TT	265 (59.8)	207 (61.8)	0.149	AA *vs*. TT	0.51 [0.26–1.03]
TA	148 (33.4)	116 (34.6)		TA / AA *vs*. TT	0.92 [0.69–1.23]
AA	30 (6.8)	12 (3.6)		TT / TA *vs*. AA	0.51 [0.26–1.02]
A / T allele	208 (23.5)/ 678 (76.5)	140 (20.9)/ 530 (79.1)	0.226	A *vs*. T	0.86 [0.68–1.10]
rs235769 (G > A)				GA *vs*. GG	1.00 [0.74–1.36]
GG	274 (62.0)	212 (63.5)	0.343	AA *vs*. GG	0.60 [0.29–1.21]
GA	142 (32.1)	110 (32.9)		GA / AA *vs*. GG	0.94 [0.70–1.26]
AA	26 (5.9)	12 (3.6)		GG / GA *vs*. AA	0.60 [0.30–1.20]
A / G allele	194 (21.9)/ 690 (78.1)	134 (20.1)/ 534 (79.9)	0.368	A *vs*. G	0.89 [0.70–1.14]
rs3178250 (T > C)				TC *vs*. TT	0.74 [0.53–1.04]
TT	121(27.5)	103 (30.8)	**0.039**	CC *vs*. TT	1.15 [0.77–1.71]
TC	236 (53.5)	149 (44.6)		TC / CC *vs*. TT	0.85 [0.62–1.16]
CC	84 (19.0)	82 (24.6)		TT / TC *vs*. CC	1.38 [0.98–1.95]
C / T allele	404 (45.8)/ 478 (54.2)	313 (46.9)/ 355 (53.1)	0.681	C *vs*. T	1.04 [0.85–1.28]

^a^Two-side chi-square test for differences in the frequency distribution of the genotypes between the cases and controls

^b^OR, odds ratio; 95% CI, 95% confidence interval.

For rs15705, the distribution of AA/AC/CC was 120/236/85 among the controls and 103/150/82 among the cases, which was statistically significant (*P* = 0.046). However, its allele distributions, C/A, were 406/476 and 314/356 in the controls and the cases, respectively, which were not significantly different (*P* = 0.744). We further performed logistic regression analysis with different comparison models. Specifically, the OR and 95% CI was 0.74 [0.53–1.03] and 1.12 [0.75–1.68] for AC *vs*. AA and CC *vs*. AA under the co-dominant model, 0.84 [0.62–1.15] for AC/CC *vs*. AA under the dominant model, and 1.36 [0.96–1.91] for AA/AC *vs*. CC under the recessive model. In addition, the OR and 95% CI for C *vs*. A was 1.03 [0.85–1.27]. The association results for the other three SNPs are also presented in the same way. In sum, based on the single SNP analysis, we found that rs15705 and rs3178250 were potentially associated with an increased susceptibility to tooth agenesis, and we therefore conducted further subgroup analysis according to the position and severity of tooth agenesis.

### Subgroup analysis according to the position of tooth agenesis

The results are presented in Table 2, S4 and S5 Tables. We found significant associations between rs15705 or rs3178250 and mandibular incisor agenesis. As shown in Table 2, the distribution of rs15705 AA/AC/CC was 120/236/85 among the controls and 52/89/53 among the cases, such that this SNP shows a significant association with mandibular incisor agenesis (AA/AC *vs*. CC = 1.58, 95% CI = [1.06–2.34], *P* = 0.024). Similar results were found for rs3178250. Compared with the rs3178250 TT/TC genotype, the rs3178250 CC genotype contributes to a higher risk of mandibular incisor agenesis (TT/TC *vs*. CC = 1.60, 95% CI = [1.08–2.37], *P* = 0.020).

**Table 2:** Association of *BMP2* SNPs and risk of mandibular incisor agenesis.

Genotype	Controls	Mandibular incisor agenesis	Pattern	*P*^a^	OR (95% CI) ^b^
	*N* = 444 (%)	*N* = 194 (%)			
rs15705 (A > C)			AC *vs*. AA	0.503	0.87 [0.58–1.31]
AA	120 (27.2)	52 (26.8)	CC *vs*. AA	0.131	1.44 [0.90–2.31]
AC	236 (53.5)	89 (45.9)	AC / CC *vs*. AA	0.915	1.02 [0.70–1.50]
CC	85 (19.3)	53 (27.3)	AA / AC *vs*. CC	**0.024**	**1.58 [1.06–2.34]**
C/A allele	406 (46.0)/ 476 (54.0)	195 (50.3)/ 193 (49.7)	A *vs*. C	0.165	1.19 [0.93–1.50]
rs3178250 (T > C)			TC *vs*. TT	0.528	0.88 [0.59–1.32]
TT	121 (27.5)	52 (26.8)	CC *vs*. TT	0.111	1.47 [0.92–2.36]
TC	236 (53.5)	89 (45.9)	TC / CC *vs*. TT	0.869	1.03 [0.71–1.51]
CC	84 (19.0)	53 (27.3)	TT / TC *vs*. CC	**0.020**	**1.60 [1.08–2.37]**
C / T allele	404 (45.8)/ 478 (54.2)	195 (50.3)/ 193 (49.7)	T *vs*. C	0.143	1.20 [0.94–1.52]

^a^ Two-sided chi-square test

^b^ OR, odds ratio; 95% CI, 95% confidence interval.

There were no significant associations between rs15705 or rs3178250 and other types of congenitally missing teeth. Detailed information is shown in S4 and S5 Tables.

### Subgroup analysis according to the severity of tooth agenesis

According to the severity of tooth agenesis, all cases were divided into two groups: cases with three or fewer absent teeth, and cases with more than three absent teeth. However, as shown in S6 Table, no significant associations were observed.

Moreover, we performed haplotype analysis on these four SNPs. However, none of the haplotypes (CTGC, ATGT and AAAT) was found to be significantly associated with susceptibility to tooth agenesis in either the overall or subgroup analysis (S7 Table).

### Functional studies on rs15705 and rs3178250

Bioinformatics analysis with four databases (Targetscan: http://www.targetscan.org/, miRanda: http://www.microrna.org/, MirSNP: http://bioinfo.bjmu.edu.cn/mirsnp/search/ and miRDB: http://www.mirdb.org/miRDB/) showed that rs15705 is located within the potential binding site of miR-1273d. An A to C alternation could thus contribute to an increase in the binding of target miRNAs. A similar effect was observed on rs3178250 and miR-4639-5p, in which the rs3178250 T allele to C allele alternation could contribute to an increased binding ability to the target miRNAs. Therefore, these predictions provide a possible underlying mechanism for how these SNPs contribute to increasing the susceptibility to tooth agenesis.

**Fig 2:**
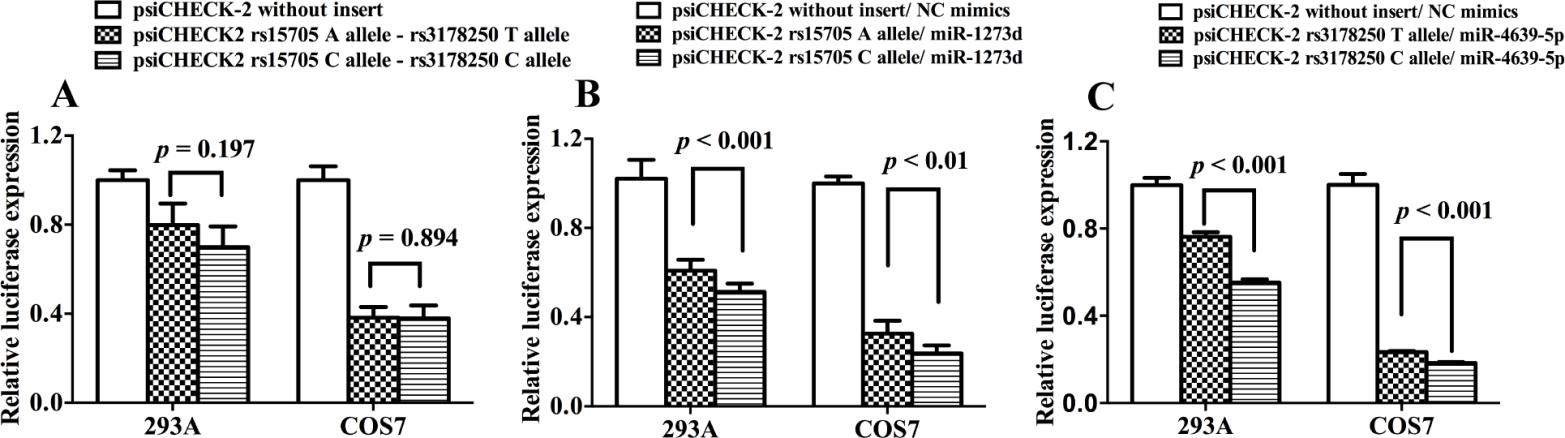
The renilla-to-firefly luminescence ratio comparison when co-transfecting 293A and COS7 cells with the *BMP2* 3’-UTR reporter and miR-mimics. PsiCHECK-2 without the insert or with the WT or MT plasmids was transfected respectively (A) or co-transfected with the miR-1273d mimics (B) or miR-4639-5p mimics (C) in the 293A and COS7 cell lines. Data were derived from three independent experiments with three replicates.

**Fig 3:**
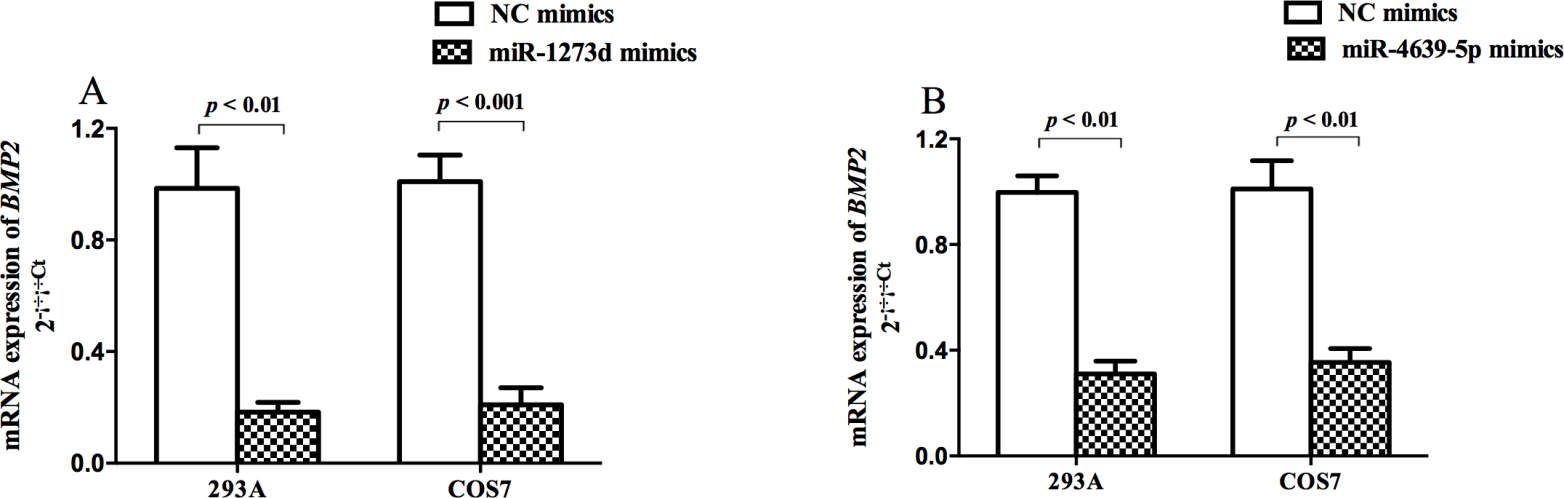
*BMP2* mRNA expression in cells after transfenction with miRNA mimics. MiR-1273d mimics (A) or miR-4639-5p mimics (B) were transfected into 293A or COS7 cells. Transcript levels were analyzed by qPCR and normalized to *GAPDH* levels. Error bars indicate the +SD obtained from three independent experiments following three replicates.

To test these predictions, the 1174bp *BMP2* 3’-UTR region with wild type (WT) or mutation type (MT) was cloned into the psiCHECK^TM^-2 vector to construct the plasmids and perform a luciferase reporter assay. Control, WT or MT plasmids and their respective miRNAs were co-transfected into 293A cells and COS7 cells. As shown in Fig 2A, the rs15705 A and C alleles contributed to significantly different relative luciferase expression in the two cell lines (*P* < 0.001 in 293A and *P* < 0.01 in COS7), indicating their possible differential binding efficiency with miR-1273d. Similarly, rs3178250 might also be able to affect the binding ability between *BMP2* 3’-UTR and miR-4639-5p in both cell lines (*P* < 0.001 in 293A and *P* < 0.001 in COS7, Fig 2B). Similar results were observed when co-transfected with 10pmol, 20pmol or 40pmol miR-mimics and psiCHECK-2 plasmids (S2 Fig). Furthermore, we found that either miR-1273d or miR-4639-5p, when transfected into these two cell lines, resulted in decreased *BMP2* mRNA expression (*P* < 0.01 in 293A and *P* < 0.001 in COS7 for miR-1273d, and *P* < 0.01 in both cell lines for miR-4639-5p). This further confirms the potential interaction between these two miRNAs and *BMP2* 3’-UTR (Fig 3).

## Discussion

*BMP2* is important for tooth development. As a member of the *BMP* pathway, *BMP2* acts from a distance to influence cell behavior by interacting with other pathways, such as the Wnt/β-catenin signaling pathway [21]. Mutations and genetic variants of this pathway, such as *WNT10A*, *AXIN2* and *LRP6*, have all been well recognized as susceptibility factors of tooth agenesis [24–26]. *BMP2* is a downstream target of *MSX1* in the dental epithelium and mesenchyme. *MSX1* can inhibit its expression to promote proliferation and prevent the differentiation of dental mesenchymal cells, thereby contributing to tooth agenesis [27]. The interaction between *BMP2/7* and the p38α MAPK pathway is also critical for the morphogenesis of tooth cusps and the secretion of dental enamel [28]. In addition, *BMP2* plays important roles in the development of incisors [29]. Therefore, genetic variants of *BMP2* are potentially susceptibility factors for non-syndromic tooth agenesis.

Several studies have previously been conducted to investigate this issue. For instance, two mutations of *BMP2* were detected in two Mexican families with tooth agenesis [30]. In Chinese populations, as far as we know, two studies have focused on genetic variations of *BMP2* and the risk of tooth agenesis. Liu H and his colleges found two SNPs (rs3178250 and rs235768) of *BMP2* with no association with tooth agenesis [31]. Nevertheless, in another study, one novel *BMP2* gene mutation was found in association with tooth agenesis [32]. Thus, these studies provided us with a limited yet conflicted understanding of the genetic contributions of *BMP2* in the development of tooth agenesis, which inspired us to conduct the present study.

In our study, four potentially functional SNPs (rs15705, rs235768, rs235769, and rs3178250) in the *BMP2* gene were chosen and investigated in a case-control study of 335 tooth agenesis cases and 444 controls to clarify their associations with non-syndromic tooth agenesis as well as specific types and severity of tooth agenesis. Our results showed that rs15705 and rs3178250, located at the 3′-UTR of the *BMP2* gene, are potentially associated with non-syndromic tooth agenesis. In the following stratified analysis, both rs15705 and rs3178250 showed associations with mandibular incisor agenesis. However, we did not find any associations with the severity of tooth agenesis.

Furthermore, bioinformatics analysis and our *in vitro* studies indicated that the mutant allele of rs15705 and rs3178250 were likely to increase binding ability of miRNAs, thereby resulting in the decreased expression of *BMP2*. In addition, the previous studies also indicated that rs15705 is associated with different protein binding affinities and a higher mRNA decay rate compared to normal sequence [33–34]. Although such *in vitro* functional studies cannot fully represent the true *in vivo* scenario, they suggest the possibility that these two SNPs might modify the expression of *BMP2* during tooth development and impose potential effects on the biological processes in which *BMP2* is involved in, contributing to the failure of tooth development.

Two major limitations of the present study should be addressed. First of all, our associations were border-line, in that they were not strong enough to withstand multiple corrections. Nevertheless, our study, along with functional studies by Fritz *et al*. and Devaney *et al*. have consistently indicated that these associated SNPs are functional; therefore, it is quite possible that these associations are genuine. In addition, based on our sample size, we still had 76.5% power to achieve these results. That said, further replication studies will be required to verify our findings. Secondly, further functional studies on the SNPs, assessing their influence on the *BMP2* protein levels as well as the other genes involved in the *BMP2* pathway, will be warranted to clarify their functional significance.

Taken together, our study indicated that rs15705 and rs3178250, located at the *BMP2* 3’-UTR and potentially affecting the miRNA-mRNA interaction, are associated with an increased risk of non-syndromic tooth agenesis. These findings help to enrich our understanding of the etiology of non-syndromic tooth agenesis.

## Supporting Information

S1 Fig Details of the *BMP2* 3’-UTR plasmids. (TIF)

S2 Fig The relative luciferase expression when transfected with different concentrations of miRNA mimics. A is for miR-1273d; B is for miR-4639-5p (*: *P* < 0.05, **: *P* < 0.01, ***: *P* < 0.001).
(TIFF)

S1 Table Sequence of the TaqMan probes and primer. (DOC)

S2 Table Basic Information on the selected SNPs of *BMP2*. (DOC)

S3 Table Characteristics of the tooth agenesis cases and controls. (DOC)

S4 Table Associations of the *BMP2* SNPs with mandibular tooth agenesis. (DOC)

S5 Table Associations of *BMP2* SNPs with maxillary tooth agenesis. (DOC)

S6 Table Associations of *BMP2* SNPs with the severity of tooth agenesis. (DOC)

S7 Table Analysis of the *BMP2* haplotypes between the controls and cases. (DOC)

## References

[1] Khalaf K, Miskelly J, Voge E, Macfarlane TV. Prevalence of hypodontia and associated factors: a systematic review and meta-analysis. J orthod. 2014; 41(4): 299–316. 10.1179/1465313314Y.0000000116 25404667

[2] Zhang J, Liu HC, Lyu X, Shen GH, Deng XX, Li WR, et al Prevalence of tooth agenesis in adolescent Chinese populations with or without orthodontics. Chin J Dent Res. 2015; 18(1): 59–65. 25815384

[3] Polder BJ, Van't Hof MA, Van der Linden FP, Kuijpers-Jagtman AM. A meta-analysis of the prevalence of dental agenesis of permanent teeth. Community Dent Oral Epidemiol. 2004; 32(3): 217–226. 15151692 10.1111/j.1600-0528.2004.00158.x

[4] Acevedo AC, Poulter JA, Alves PG, de Lima CL, Castro LC, Yamaguti PM, et al Variability of systemic and oro-dental phenotype in two families with non-lethal Raine syndrome with mutations. BMC Med Genet. 2015; 16 (1): 8 10.1186/s12881-015-0154-5 25928877 PMC4422040

[5] Lai LH, Hui BK, Nguyen PD, Yee KS, Martz MG, Bradley JP, et al Lateral incisor agenesis predicts maxillary hypoplasia and Le Fort I advancement surgery in cleft patients. Plast Reconstr Surg. 2015; 135(1): 142e–148e. 10.1097/PRS.0000000000000779 25539321

[6] Alshaiji JM, Handler MZ, Huo R, Freedman A, Schachner LA. HATS syndrome: hemimaxillary enlargement, asymmetry of the face, tooth abnormalities, and skin findings. Cutis. 2014; 94(4): E18–21. 25372264

[7] Lyngstadaas SP, Nordbo H, Gedde-Dahl T Jr, Thrane PS. On the genetics of hypodontia and microdontia: synergism or allelism of major genes in a family with six affected members. J Med Genet. 1996; 33(2): 137–142. 8929951 10.1136/jmg.33.2.137 PMC1051840

[8] Lopez SI, Mundstock KS, Paixão-Côrtes VR, Schüler-Faccini L, Mundstock CA, Bortolini MC, et al MSX1 and PAX9 investigation in monozygotic twins with variable expression of tooth agenesis. Twin Res Hum Genet. 2013, 16(6): 1112–1116. 10.1017/thg.2013.69 24103583

[9] Halicioglu K, Sahin H, Corekci B, Irgin C, Toptas O. Isolated oligodontia in monozygotic twins. Eur J Dent. 2013; 7(Suppl 1): S111–1114. 10.4103/1305-7456.119087 24966717 PMC4054068

[10] Mostowska A, Biedziak B, Jagodzinski PP. Novel MSX1 mutation in a family with autosomal-dominant hypodontia of second premolars and third molars. Arch Oral Biol. 2012; 57(6): 790–795. 10.1016/j.archoralbio.2012.01.003 22297032

[11] Botstein D, Risch N. Discovering genotypes underlying human phenotypes: past successes for mendelian disease, future approaches for complex disease. Nat Genet. 2003; 33 (Suppl): 228–237. 12610532 10.1038/ng1090

[12] Haga S, Nakaoka H, Yamaguchi T, Yamamoto K, Kim YI, Samoto H, et al A genome-wide association study of third molar agenesis in Japanese and Korean populations. J Hum Genet. 2013; 58(12): 799–803. 10.1038/jhg.2013.106 24172245

[13] Song S, Zhao R, He H, Zhang J, Feng H, Lin L. WNT10A variants are associated with non-syndromic tooth agenesis in the general population. Hum Genet. 2014; 133(1): 117–124. 10.1007/s00439-013-1360-x 24043634

[14] Eichler EE, Flint J, Gibson G, Kong A, Leal SM, Moore JH, et al Missing heritability and strategies for finding the underlying causes of complex disease. Nat Rev Genet. 2010; 11(6): 446–450. 10.1038/nrg2809 20479774 PMC2942068

[15] Saadi I, Das P, Zhao M, Raj L, Ruspita I, Xia Y, et al Msx1 and Tbx2 antagonistically regulate Bmp4 expression during the bud-to-cap stage transition in tooth development. Development. 2013; 140 (13): 2697–2702. 10.1242/dev.088393 23720046 PMC3678339

[16] Gong M, Qian YJ, Gu N, Wang W, Wang H, Ma L, et al Association of BMP4 polymorphisms with isolated tooth agenesis in a Chinese Han population: a case-control study. Eur Rev Med Pharmacol Sci. 2015; 19(12): 2188–2194. 26166641

[17] Zhang YD, Chen Z, Song YQ, Liu C, Chen YP. Making a tooth: growth factors, transcription factors, and stem cells. Cell Res. 2005; 15(5): 301–316. 15916718 10.1038/sj.cr.7290299

[18] Tasli PN, Aydin S, Yalvac ME, Sahin F. Bmp 2 and bmp 7 induce odonto- and osteogenesis of human tooth germ stem cells. Appl Biochem Biotech. 2014; 172(6): 3016–3025. 10.1007/s12010-013-0706-0 24477555

[19] Suzuki T, Bessho K, Segami N, Iizuka T, Nojima T. Immunohistochemical localization of bone morphogenetic protein-2 in the oral and maxillofacial area of the human embryo. Br J Oral Maxillofac Surg. 2001; 39(4): 289–293. 11437427 10.1054/bjom.2000.0568

[20] Neubuser A, Peters H, Balling R, Martin GR. Antagonistic interactions between FGF and BMP signaling pathways: a mechanism for positioning the sites of tooth formation. Cell. 1997; 90(2): 247–255. 9244299 10.1016/s0092-8674(00)80333-5

[21] Yuan G, Yang G, Zheng Y, Zhu X, Chen Z, Zhang Z, et al The non-canonical BMP and Wnt/beta-catenin signaling pathways orchestrate early tooth development. Development. 2015; 142(1): 128–139. 10.1242/dev.117887 25428587 PMC4299140

[22] Iohara K, Nakashima M, Ito M, Ishikawa M, Nakasima A, Akamine A. Dentin regeneration by dental pulp stem cell therapy with recombinant human bone morphogenetic protein 2. J Dent Res. 2004; 83(8): 590–595. 15271965 10.1177/154405910408300802

[23] Ma L, Xu M, Li D, Han Y, Wang Z, Yuan H, et al A miRNA-binding-site SNP of MSX1 is Associated with NSOC Susceptibility. J Dent Res. 2014; 93(6): 559–564. 10.1177/0022034514527617 24603642

[24] Song S, Zhao R, He H, Zhang J, Feng H, Lin L. WNT10A variants are associated with non-syndromic tooth agenesis in the general population. Hum Genet. 2014; 133(1): 117–124. 10.1007/s00439-013-1360-x 24043634

[25] Mu YD, Xu Z, Contreras CI, McDaniel JS, Donly KJ, Chen S. Mutational analysis of AXIN2, MSX1, and PAX9 in two Mexican oligodontia families. Genet Mol Res., 2013; 12(4): 4446–4458. 10.4238/2013 24222224 PMC3977612

[26] Massink MP1 Créton MA, Spanevello F, Fennis WM, Cune MS, Savelberg SM, et al Loss-of-Function Mutations in the WNT Co-receptor LRP6 Cause Autosomal-Dominant Oligodontia. Am J Hum Genet, 2015; 97(4): 621–626. 10.1016/j.ajhg.2015.08.014 26387593 PMC4596913

[27] Feng XY, Zhao YM, Wang WJ, Ge LH. Msx1 regulates proliferation and differentiation of mouse dental mesenchymal cells in culture. Eur J Oral Sci. 2013; 121(5): 412–420. 10.1111/eos.12078 24028588

[28] Greenblatt MB, Kim JM, Oh H, Park KH, Choo MK, Sano Y, et al p38alpha MAPK is required for tooth morphogenesis and enamel secretion. J Biol Chem. 2015; 290(1): 284–295. 10.1074/jbc.M114.599274 25406311 PMC4281732

[29] Vogel P, Liu J, Platt KA, Read RW, Thiel M, Vance RB, et al Malformation of incisor teeth in Grem2 (-)/ (-) mice. Vet Pathol. 2015; 52(1): 224–229. 10.1177/0300985814528218 24686385

[30] Mu Y, Xu Z, Contreras CI, McDaniel JS, Donly KJ, Chen S. Phenotype characterization and sequence analysis of BMP2 and BMP4 variants in two Mexican families with oligodontia. Genet Mol Res. 2012; 11(4): 4110–4120. 10.4238/2012 23079991 PMC3569492

[31] Liu H, Zhang J, Song S, Zhao H, Han D, Feng H. A case-control study of the association between tooth-development gene polymorphisms and non-syndromic hypodontia in the Chinese Han population. Eur J Oral Sci. 2012; 120(5): 378–385. 10.1111/j.1600-0722.2012.00986.x 22984994

[32] Zou C, Gao QP, Hussam HB, Wang W, Bai XN, He FQ. BMP2/BMP4 genetic evaluation in 40 patients with tooth agenesis. Shanghai Kou Qiang Yi Xue. 2015; 24(1): 83–88. 25858375

[33] Fritz DT, Jiang S, Xu J, Rogers MB. A polymorphism in a conserved posttranscriptional regulatory motif alters bone morphogenetic protein 2 (BMP2) RNA: protein interactions. Mol Endocrinol. 2006; 20(7): 1574–1586. 16497730 10.1210/me.2005-0469

[34] Devaney JM, Tosi LL, Fritz DT, Gordish-Dressman HA, Jiang S, Orkunoglu-Suer FE, et al Differences in fat and muscle mass associated with a functional human polymorphism in a post-transcriptional BMP2 gene regulatory element. J Cell Biochem. 2009; 107(6): 1073–1082. 10.1002/jcb.22209 . 19492344 PMC4147943

