# Supplementary figures and images for: Genetic Variants of BMP2 and Their Association with the Risk of Non-Syndromic Tooth Agenesis
Source: PLoS One. 2016 Jun 30;11(6):e0158273. doi: 10.1371/journal.pone.0158273 (PMC4928851; doi:10.1371/journal.pone.0158273)

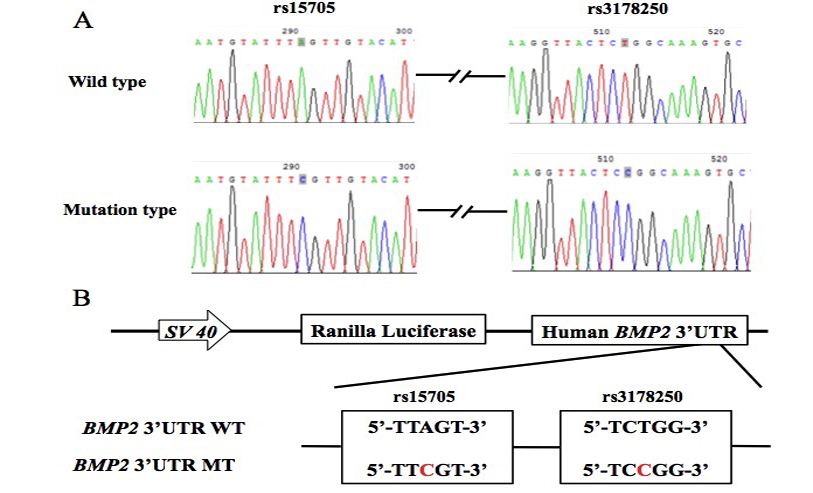

Supplement: S1 Fig — (TIF) [file pone.0158273.s001.tif]

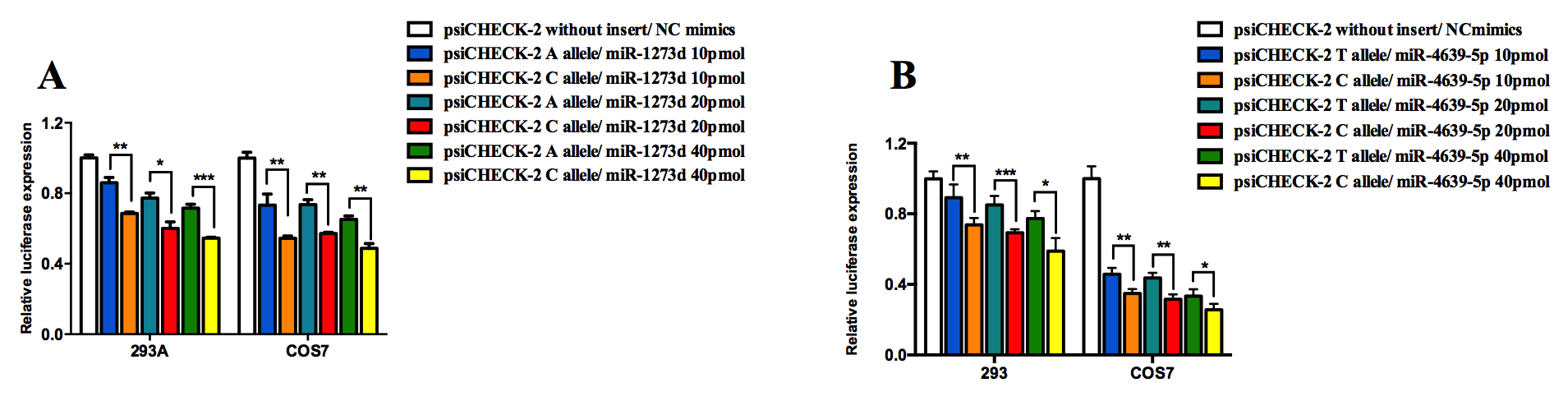

Supplement: S2 Fig — A is for miR-1273d; B is for miR-4639-5p (*: P < 0.05, **: P < 0.01, ***: P < 0.001). (TIFF) [file pone.0158273.s002.tiff]
