# Supplementary material for: Genetic Variants of BMP2 and Their Association with the Risk of Non-Syndromic Tooth Agenesis
Source: PLoS One. 2016 Jun 30;11(6):e0158273. doi: 10.1371/journal.pone.0158273 (PMC4928851; doi:10.1371/journal.pone.0158273)
Supplement: S1 Table — (DOC) [file pone.0158273.s003.doc]

| **SNP** | **Primer (5’-3’)** | **Probe (5’-3’-MGB)** |
| --- | --- | --- |
| rs15705 | F:AATAAATCTGACCATTATACTTCATGTGCT | T:FAM-AAAATGTACAACTAAATAC |
|  | R:ATATCTACGAAAAGAAGTTGGGAAAACA | G:HEX-AATGTACAACGAAATAC |
| rs235768 | F:CAATAGCAGTTTCCATCACCGAATT | A:FAM-TCCAAAAGACTGGTCAC |
|  | R:GTGACATCAAAACTTTCCCACCTG | T:HEX-TCCAAAAGTCTGGTCAC |
| rs235769 | F:CCACTCTGCTGACTTTCAAGATTATT | G:FAM-TGCAGAGTGGTTGTC |
|  | R:AGCAATGTCTGGTTCTTATCCAAAT | A:HEX-CAGAGTGATTGTCCAAT |
| rs3178250 | F:AAGTATCGGGTTTGTACATAATTTTCC | T:FAM-ATGGAAGGTTACTCTGG |
|  | R:TGCAAAAAAGCAAACGTGCTA | C:HEX-ATGGAAGGTTACTCCGG |

**S1 Table.** **Sequence of the TaqMan probes and primer**
