# Supplementary material for: Genetic Variants of BMP2 and Their Association with the Risk of Non-Syndromic Tooth Agenesis
Source: PLoS One. 2016 Jun 30;11(6):e0158273. doi: 10.1371/journal.pone.0158273 (PMC4928851; doi:10.1371/journal.pone.0158273)
Supplement: S2 Table — (DOC) [file pone.0158273.s004.doc]

**S2 Table. Basic Information on the selected SNPs of *BMP2***

| **SNP ID** | **Chromosome Position** | **Gene location** | **Base (major>minor)** | **MAF^a^ CHB^b^** | **Call Rate (%) (control / case)** | **HWE^c^ among controls** |
| --- | --- | --- | --- | --- | --- | --- |
| rs15705 | 20:6779333 | 3’UTR | A > C | C 0.442 | 99.32 / 100.00 | 0.11 |
| rs235768 | 20:6778468 | Exon | T > A | A 0.261 | 99.77 / 100.00 | 0.14 |
| rs235769 | 20:6779697 | 3’UTR | G > A | A 0.217 | 99.55 / 99.70 | 0.19 |
| rs3178250 | 20:6779554 | 3’UTR | T > C | C 0.488 | 99.32 / 99.70 | 0.10 |

MAF^a^: minor allele frequency

CHB^b^: Han Chinese in Beijing

HWE^c^: Hardy-Weinberg equilibrium
