# Supplementary material for: Genetic Variants of BMP2 and Their Association with the Risk of Non-Syndromic Tooth Agenesis
Source: PLoS One. 2016 Jun 30;11(6):e0158273. doi: 10.1371/journal.pone.0158273 (PMC4928851; doi:10.1371/journal.pone.0158273)
Supplement: S3 Table — (DOC) [file pone.0158273.s005.doc]

**S3 Table. Characteristics of the** tooth agenesis cases and controls

|  | **Controls** | **Cases** |  |
| --- | --- | --- | --- |
|  | *N* = 444 (%) | *N* = 335 (%) | *P* |
| Gender |  |  |  |
| Male | 155 (34.9) | 123 (36.7) | 0.602^a^ |
| Female | 289 (65.1) | 212 (63.3) |  |
| Age |  |  |  |
| (Mean ± SD) | 17.05 ± 8.34 | 16.42 ± 6.57 | 0.265^b^ |

^a^Chi-Squeare test

^b^Independent-Sample *t* Test
