# Supplementary material for: Genetic Variants of BMP2 and Their Association with the Risk of Non-Syndromic Tooth Agenesis
Source: PLoS One. 2016 Jun 30;11(6):e0158273. doi: 10.1371/journal.pone.0158273 (PMC4928851; doi:10.1371/journal.pone.0158273)
Supplement: S4 Table — (DOC) [file pone.0158273.s006.doc]

**S4 Table. Associations of *BMP2* SNPs with the** tooth agenesis in mandibular subgroups

| **Genotype** | **Controls** | **Mandibular canine agenesis** | **OR (95%CI) ^a^** | **Mandibular premolar agenesis** | **OR (95%CI) ^a^** | **Mandibular molar agenesis** | **OR (95%CI) ^a^** |
| --- | --- | --- | --- | --- | --- | --- | --- |
| rs15705 (A > C) | N = 444 (%) | N = 9 (%) |  | N = 69 (%) |  | N = 4 (%) |  |
| AA | 120 (27.2) | 2 (22.2) | 1.00 | 25 (36.2) | 1.00 | 1 (25.0) | 1.00 |
| AC | 236 (53.5) | 3 (33.3) | 0.76 [0.13-4.63] | 30 (43.5) | 0.61 [0.34-1.08] | 2 (50.0) | 1.02 [0.09-11.33] |
| CC | 85 (19.3) | 4 (44.5) | 2.82 [0.51-15.77] | 14 (20.3) | 0.79 [0.39-1.61] | 1 (25.0) | 1.41 [0.08-22.89] |
| AC / CC *vs* AA | 321 (72.8) | 7 (77.8) | 1.31 [0.27-6.39] | 44 (63.8) | 0.66 [0.39-1.12] | 3 (60.0) | 1.12 [0.11-10.89] |
| AA / AC *vs* CC | 356 (80.7) | 5 (55.6) | 3.35 [0.88-12.74] | 55 (79.7) | 1.07 [0.57-2.01] | 3 (60.0) | 1.40 [0.14-13.59] |
| C / A allele | 406 (46.0)/  476 (54.0) | 7 (38.9)/  11 (61.1) | 1.84 [0.71-4.80] | 58 (42.0)/  80 (58.0) | 0.85 [0.59-1.22] | 4 (50.0)/  4 (50.0) | 1.17 [0.29-4.72] |
| rs3178250 (T > C) | N = 444 (%) | N = 9 (%) |  | N = 69 (%) |  | N = 4 (%) |  |
| TT | 121 (27.5) | 2 (22.2) | 1.00 | 25 (36.2) | 1.00 | 1 (25.0) | 1.00 |
| TC | 236 (53.5) | 3 (33.3) | 0.77 [0.13-4.67] | 30 (43.5) | 0.62 [0.35-1.09] | 2 (50.0) | 1.03 [0.09-11.42] |
| CC | 84 (19.0) | 4 (44.5) | 2.88 [0.52-16.09] | 14 (20.3) | 0.81 [0.40-1.64] | 1 (25.0) | 1.44 [0.09-23.35] |
| TC / CC *vs* TT | 320 (72.6) | 7 (77.8) | 1.32 [0.27-6.46] | 44 (63.8) | 0.67 [0.39-1.14] | 3 (60.0) | 1.13 [0.12-11.01] |
| TT / TC *vs* CC | 357 (81.0) | 5 (55.6) | 3.40 [0.89-12.93] | 55 (79.7) | 1.08 [0.57-2.04] | 3 (60.0) | 1.42 [0.15-13.79] |
| C / T allele | 404 (45.8)/ 478 (54.2) | 7 (38.9)/  11 (61.1) | 1.86 [0.71-4.84] | 58 (42.0)/  80 (58.0) | 0.86 [0.60-1.23] | 4 (50.0)/  4 (50.0) | 1.18 [0.29-4.76] |

^a^ OR, odds ratio; 95% CI, 95% confidence interval.
