# Supplementary material for: Genetic Variants of BMP2 and Their Association with the Risk of Non-Syndromic Tooth Agenesis
Source: PLoS One. 2016 Jun 30;11(6):e0158273. doi: 10.1371/journal.pone.0158273 (PMC4928851; doi:10.1371/journal.pone.0158273)
Supplement: S5 Table — (DOC) [file pone.0158273.s007.doc]

| **Genotype** | **Controls** | **Maxillary incisor agenesis** | **OR (95%CI) ^a^** | **Maxillary canine agenesis** | **OR (95%CI) ^a^** | **Maxillary premolar agenesis** | **OR (95%CI) ^a^** | **Maxillary molar agenesis** | **OR (95%CI) ^a^** |
| --- | --- | --- | --- | --- | --- | --- | --- | --- | --- |
| rs15705 (A > C) | N = 444 (%) | N = 56 (%) |  | N = 33 (%) |  | N = 42 (%) |  | N = 5 (%) |  |
| AA | 120 (27.2) | 19 (33.9) | 1.00 | 7 (21.2) | 1.00 | 16 (38.1) | 1.00 | 1 (20.0) | 1.00 |
| AC | 236 (53.5) | 25 (44.6) | 0.67 [0.35-1.26] | 18 (54.6) | 1.31 [0.53-3.22] | 17 (40.5) | 0.54 [0.26-1.11] | 2 (40.0) | 1.02 [0.09-11.33] |
| CC | 85 (19.3) | 12 (21.4) | 0.89 [0.41-1.03] | 8 (24.2) | 1.61 [0.56-4.62] | 9 (21.4) | 0.79 [0.34-1.88] | 2 (40.0) | 2.82 [0.25-31.64] |
| AC / CC *vs* AA | 321 (72.8) | 37 (66.1) | 0.73 [0.40-1.32] | 26 (78.8) | 1.39 [0.59-3.28] | 26 (61.9) | 0.61 [0.32-1.17] | 4 (80.0) | 1.50 [0.17-13.51] |
| AA / AC *vs* CC | 356 (80.7) | 44 (78.6) | 1.14 [0.58-2.26] | 25 (75.8) | 1.34 [0.58-3.08] | 33 (78.6) | 1.14 [0.53-2.45] | 3 (60.0) | 2.79 [0.46-16.97] |
| C/A allele | 406 (46.0)/  476 (54.0) | 49 (43.7)/  63 (56.3) | 0.91 [0.61-1.36] | 34 (51.5)/  32 (48.5) | 1.25 [0.76-2.06] | 35 (41.7)/  49 (58.3) | 0.84 [0.52-1.32] | 4 (40.0)/  6 (60.0) | 1.76 [0.49-6.28] |
| rs3178250 (T > C) | N = 444 (%) | N = 56 (%) |  | N = 33 (%) |  | N = 42 (%) |  | N = 5 (%) |  |
| TT | 121 (27.5) | 19 (34.6) | 1.00 | 7 (21.2) | 1.00 | 16 (38.1) | 1.00 | 1 (20.0) | 1.00 |
| TC | 236 (53.5) | 24 (43.6) | 0.65 [0.34-1.23] | 18 (54.6) | 1.32 [0.54-3.24] | 17 (40.5) | 0.55 [0.27-1.12] | 2 (40.0) | 1.03 [0.09-11.42] |
| CC | 84 (19.0) | 12 (21.8) | 0.91 [0.42-1.97] | 8 (24.2) | 1.65 [0.58-4.71] | 9 (21.4) | 0.81 [0.34-1.92] | 2 (40.0) | 2.88 [0.26-32.29] |
| TC / CC *vs* TT | 320 (72.6) | 36 (65.5) | 0.72 [0.40-1.30] | 26 (78.8) | 1.40 [0.59-3.32] | 26 (61.9) | 0.61 [0.32-1.19] | 4 (80.0) | 1.51 [0.17-13.67] |
| TT / TC *vs* CC | 357 (81.0) | 43 (78.2) | 1.18 [0.60-2.35] | 25 (75.8) | 1.36 [0.59-3.12] | 33 (78.6) | 1.16 [0.53-2.51] | 3 (60.0) | 2.83 [0.47-17.23] |
| C / T allele | 404 (45.8)/ 478 (54.2) | 48 (43.6)/  62 (56.4) | 0.92 [0.61-1.37] | 34 (51.5)/  32 (48.5) | 1.26 [0.76-2.07] | 35 (41.7)/  49 (58.3) | 0.85 [0.54-1.33] | 4 (40.0)/  6 (60.0) | 1.78 [0.50-6.33] |

**S5 Table**. **Associations of *BMP2* SNPs and tooth agenesis in maxillary subgroups**

^a^ OR, odds ratio; 95% CI, 95% confidence interval.
