# Supplementary material for: Genetic Variants of BMP2 and Their Association with the Risk of Non-Syndromic Tooth Agenesis
Source: PLoS One. 2016 Jun 30;11(6):e0158273. doi: 10.1371/journal.pone.0158273 (PMC4928851; doi:10.1371/journal.pone.0158273)
Supplement: S6 Table — (DOC) [file pone.0158273.s008.doc]

**S6 Table. Associations of *BMP2* SNPs and the severity of tooth agenesis**

| **Genotype** | **Controls** | **1 - 3** | ***P* ^a^** | **OR (95%CI) ^b^** | **> 3** | ***P* ^a^** | **OR (95%CI) ^b^** |
| --- | --- | --- | --- | --- | --- | --- | --- |
| **rs15705 (A > C)** | N = 444 (%) | N = 312 (%) |  |  | N = 23 (%) |  |  |
| AA | 120 (27.2) | 98 (31.4) |  | 1.00 | 5 (21.7) |  | 1.00 |
| AC | 236 (53.5) | 140 (44.9) | 0.064 | 0.73 [0.52-1.02] | 10 (43.5) | 0.976 | 1.02 [0.34-3.04] |
| CC | 85 (19.3) | 74 (23.7) | 0.760 | 1.07 [0.71-1.61] | 8 (34.8) | 0.156 | 2.26 [0.71-7.14] |
| AC / CC *vs* AA | 321 (72.8) | 214 (68.6) | 0.211 | 0.82 [0.59-1.12] | 18 (78.3) | 0.564 | 1.35 [0.49-3.71] |
| AA / AC *vs* CC | 356 (80.7) | 238 (76.3) | 0.141 | 1.30 [0.92-1.85] | 15 (65.2) | 0.070 | 2.23 [0.92-5.44] |
| C / A allele | 406 (46.0)/  476 (54.0) | 288 (46.2)/  336 (53.8) | 0.966 | 1.00 [0.82-1.23] | 26 (56.5)/  20 (43.5) | 0.164 | 1.52 [0.84-2.77] |
| rs3178250 (T > C) | N = 444 (%) | N = 312 (%) |  |  | N = 23 (%) |  |  |
| TT | 121 (27.5) | 98 (31.5) |  | 1.00 | 5 (21.7) |  | 1.00 |
| TC | 236 (53.5) | 139 (44.7) | 0.065 | 0.73 [0.52-1.02] | 10 (43.5) | 0.964 | 1.03 [0.34-3.07] |
| CC | 84 (19.0) | 74 (23.8) | 0.688 | 1.09 [0.72-1.64] | 8 (34.8) | 0.145 | 2.31 [0.73-7.29] |
| TC / CC *vs* TT | 320 (72.6) | 213 (68.5) | 0.226 | 0.82 [0.60-1.13] | 18 (78.3) | 0.549 | 1.36 [0.49-3.75] |
| TT / TC *vs* CC | 357 (81.0) | 237 (76.2) | 0.116 | 1.33 [0.93-1.89] | 15 (65.2) | 0.065 | 2.27 [0.93-5.52] |
| C / T allele | 404 (45.8)/ 478 (54.2) | 287 (46.1)/  335 (53.9) | 0.897 | 1.01 [0.83-1.25] | 26 (56.5)/  20 (43.5) | 0.155 | 1.54 [0.85-2.80] |

^a^ Two-sided chi-square test

^b^ OR, odds ratio; 95% CI, 95% confidence interval.
