# Supplementary material for: Genetic Variants of BMP2 and Their Association with the Risk of Non-Syndromic Tooth Agenesis
Source: PLoS One. 2016 Jun 30;11(6):e0158273. doi: 10.1371/journal.pone.0158273 (PMC4928851; doi:10.1371/journal.pone.0158273)
Supplement: S7 Table — (DOC) [file pone.0158273.s009.doc]

**S7 Table. Analysis of the *BMP2* haplotypes between the** controls and cases

| **Haplotype^a^** | **Controls** | **Tooth agenesis** | **OR (95%CI)^b^** | **Mandibular incisor agenesis** | **OR (95%CI) ^b^** |
| --- | --- | --- | --- | --- | --- |
|  | *N* = 444 (%) | *N* = 335 (%) |  | *N* = 194 (%) |  |
| CTGC | 197 (44.4) | 154 (46.1) | 1.00 | 96 (49.5) | 1.00 |
| ATGT | 138 (31.0) | 110 (32.9) | 1.02 [0.74-1.42] | 61 (31.2) | 0.91 [0.62-1.34] |
| AAAT | 96 (21.6) | 67 (20.0) | 0.89 [0.61-1.30] | 36 (18.3) | 0.77 [0.49-1.21] |

^a^Order of single nucleotide polymorphisms (SNPs): rs15705- rs235768- rs235769- rs3178250.
^b^OR, odds ratio; CI, confidence interval.
